# N‐1 semi‐continuous transient perfusion in shake flask for ultra‐high density seeding of CHO cell cultures in benchtop bioreactors

**DOI:** 10.1002/btpr.70029

**Published:** 2025-04-03

**Authors:** Lucas Lemire, Sebastian‐Juan Reyes, Yves Durocher, Robert Voyer, Olivier Henry, Phuong Lan Pham

**Affiliations:** ^1^ Department of Chemical Engineering Polytechnique Montreal Montréal Quebec Canada; ^2^ Human Health Therapeutics Research Centre National Research Council Canada Montréal Quebec Canada

**Keywords:** CHO cell culture, cumate inducible system, oxygen transfer, preculture perfusion, pseudo‐perfusion, semi‐continuous perfusion, ultra‐high seeding density (UHSD)

## Abstract

One strategy to enhance the production of biological therapeutics is using transient perfusion in the preculture (N‐1 stage) to seed the production culture (N stage) at ultra‐high cell densities (>10 x 10^6^ viable cells/mL). This very high seeding density improves cell culture performance by shortening the timeline and/or achieving higher final product concentrations. Typically, an N‐1 seed train employs bioreactors with alternating tangential flow filtration (ATF) or tangential flow filtration (TFF) perfusion systems or Wave cell bag bioreactor with integrated filtration membrane, which have costs and technical complexity. Here, we propose an alternative method using semi‐continuous transient perfusion through media exchange in shake flasks, which is suitable for benchtop‐scale intensification process development. Daily media exchange was necessary to prevent nutrient limitations. The observed limitation of maximum viable cell densities (VCD) in various flask sizes was demonstrated to be due to oxygen limitations through the measurements of maximum oxygen transfer rates (OTR) using the sulfite system. By increasing agitation frequency from 200 to 300 RPM, maximum OTR in 500‐mL shake flasks was increased by 62.3%, allowing an increase in maximum VCD of 29.6%. However, in 1000‐mL shake flasks, an increase in agitation rate resulted in early cell death. After demonstrating that media exchange in shake flasks by centrifugation had no significant impact on cell growth rates, metabolism, and productivity, a benchtop bioreactor was seeded from semi‐continuous transient perfusion cell expansion. The ultra‐high cell density seeding resulted in a 49.3% increase in space–time‐yield (STY) when compared to a standard low seeding density culture.

## 1 INTRODUCTION

Biotherapeutics such as monoclonal antibodies (mAbs) are predominantly produced in large‐scale fed‐batch bioreactors with CHO cell expression systems. To supply the ever‐growing demand for these products, three options are presented: process intensification, scale‐out, and scale‐up. Scaling‐up mammalian cell cultures can be a non‐trivial task, which inherently includes risks associated with productivity and product quality.^1^ As such, alternatives to increase the production of biologics, including scale‐out and process intensification, are gaining in popularity. Scale‐out is generally more feasible than scale‐up, as it is easier to add additional bioreactors than to increase the size of a single bioreactor and can be more cost‐effective than scale‐up in terms of capital costs. This makes it easier to increase production capacity as demand grows. Process intensification, which often comes in the form of achieving high cell densities, is another method of increasing recombinant protein productivity. It has been shown that ultra‐high seeding density (UHSD) at up to 20 × 10^6^ cells/mL cell cultures to produce mAbs has enabled up to a 3‐fold increase in final titer or 75%–132% increase in space–time yield (STY) (Equation 1).^2^, ^3^, ^4^, ^5^, ^6^, ^7^, ^8^ To seed bioreactors at these elevated cell densities, cells are commonly cultured in transient perfusion mode in the N‐1 seed train vessel to satisfy the large number of cells needed.

(1) $$\mathrm{STY}=\frac{{\left[\text{Product}\right]}_{\text{final}}}{{\text{Time}}_{\text{final}}}$$

Various perfusion systems are available that could be used for the N‐1 cell expansion for UHSD cell cultures. Conventional perfusion methods utilize static, dynamic, or tangential flow (TF) coupled with membrane‐based (MB) filtration or membrane‐free (non‐MB) dedicated cell retention systems. Static MB retention systems are typically used with rocking bioreactors such as the ReadyToProcess WAVE25 and its Cellbag (Cytiva) or Biostat RM 20 and its Flexsafe RM bag (Sartorius) which aid in reducing membrane fouling through dynamic mixing. Dynamic BM retention systems such as spin and rotating disk filters have lower fouling risks. However, the moving parts housed inside the bioreactor increase contamination risks; thus, they are impractical to replace, and they are also not available in single‐use formats.^9^ To avoid fouling altogether, non‐MB methods such as acoustic filtration, centrifuge, and gravitational settlers have been used.^10^ However, these cell retention systems have downsides such as limited scalability of acoustic filters, cell damage, aggregation, and the requirement of specialized bioreactor design for centrifugation and long residence time in sub‐optimal culture conditions for gravitational settlers. Due to these limitations, acoustic filters, which were the predominant non‐MB cell retention method, have seldom been reported in the literature since 2014.^10^ TF MBs such as alternating tangential flow filtration (ATF) (Refine Technology, Repligen, and Artemis) and tangential flow filtration (TFF) which are driven by low‐shear centrifugation magnetic pumps (Levitronix) have the advantage of being kept outside of the bioreactor, allowing modularity and scalability of cell retention. For these reasons, ATF and TFF perfusion systems are the most used cell retainer devices for N‐1 perfusion for UHSD cell cultures.^10^, ^11^, ^12^

Scale‐down models of perfusion systems are necessary due to the high operating costs of benchtop bioreactor perfusion equipment and the need to optimize cell specific perfusion rates (CSPR), perfusion rate (vessel volume per day, VVD), feed and media composition, and cell line stability and scalability. These scale‐down models are typically done through semi‐continuous transient perfusion in non‐instrumented shake tubes, shake flasks, and standard and deep multi‐well plates. In 50‐mL shake tubes with fill volumes of 10–15 mL, a maximum viable cell density of 30‐70 × 10^6^ cells/mL was observed.^13^, ^14^, ^15^, ^16^ In 125‐mL baffled shake flasks with 25 mL (20%, volume/volume) fill volume, a maximum viable cell density of 45 × 10^6^ cells/mL was observed with optimized feed composition.^17^ Cell densities of up to 95 × 10^6^ cells/mL were achieved with transient semi‐continuous perfusion in 24 deep‐well plates (DWP) at working volumes of 3 mL.^18^

The impact of fill volume (5, 10, and 30 mL) in 50‐mL shake tubes was explored and it was found that larger fill volumes resulted in lower cell growth. These growth limitations are due to oxygen transfer limitations.^13^ Similarly, these oxygen limitations are expected to be observed in shake flasks. The maximum oxygen transfer rate (OTR) achievable in shake flasks depends on agitation rate, fill volume, shaking orbit diameter, flask diameter, and osmolarity levels.^19^ Increasing shake flask sizes, at the same proportional fill volume, will result in lower OTR_max_ due to volume increasing faster than flask diameter. Additionally, OTR may be an indicator of the late phase exponential growth,^20^ metabolic activity,^21^, ^22^ and cell concentration.^23^, ^24^ These metrics are of importance as bioreactors should be seeded from cells in exponential growth with high cellular metabolic activity. High cell concentration is needed in the N‐1 culture to seed the production culture (N stage). The Transfer‐Rate Online Monitoring (TOM, Kuhner Inc.) device has been shown to be useful at non‐invasively monitoring oxygen uptake rates (OUR) and carbon transfer rates (CTR) in shake flasks in order to determine critical differences in cell line behavior.^25^ Another factor that may impact oxygen transfer in shake flasks is the material of construction. The hydrophilicity of the material affects the formation of the thin liquid film during agitation. A hydrophilic material, such as glass, will form a thin layer of liquid on the surface allowing for gas transfer while wetting of a hydrophobic material, such as plastic, will not be as efficient.^26^ However, in mammalian cell cultures, such as those performed in this study, single‐use sterile shake flasks are predominantly used to minimize the risk of contamination.

In this article, we present a simple but efficient method of performing semi‐continuous transient perfusion (transient pseudo‐perfusion) in shake flasks with the objective of seeding a benchtop bioreactor for UHSD CHO cell culture production of recombinant protein. This method was developed with a cumate‐inducible CHO‐GS clonal cell line, which truly allows for a two‐phase cell culture.^27^ Using the inducible system alleviates the metabolic burden imposed on cells by protein production in the cell growth phase, thus allowing for a faster growth rate and higher achievable viable cell densities.^28^, ^29^ In the present study, we have also demonstrated, through oxygen transfer rate (OTR) measurements, that the highest viable CHO cell density was achieved in shake flasks before oxygen transfer limitations are attained.

## 2 MATERIALS AND METHODS

### 2.1 Cell culture maintenance

A proprietary stable clone of CHO‐GS cells, inducible through a cumate gene switch and capable of producing the monoclonal antibody palivizumab (PLVZM), was utilized. This cell line was internally developed, as previously detailed.^27^ Cells were cryopreserved in BalanCD CHO Growth A media (Fujifilm Irvine Scientific, USA) with 50 μM MSX and 0.1% (w/v) Kolliphor P188 (BASF, Germany) supplemented with 7.5% dimethylsulfoxide (DMSO) (Sigma Aldrich, ≥99.7% purity, USA) and stored in liquid nitrogen tanks (MVE, Series 800–190, USA) at −180 °C. For thawing, cell vials were immersed in a 37 °C water bath for a few minutes until completely thawed. Cells were expanded in shake flasks in 5% CO_2_ incubators set to 37 °C, 75% relative humidity, and 120 RPM agitation with a 25‐mm orbit. Cells were passaged every 2–3 days in growth medium consisting of BalanCD CHO Growth A and 50 μM MSX with cell density maintained between 0.2 and 3.0 × 10^6^ cells/mL.

### 2.2 Semi‐continuous transient perfusion

The semi‐continuous transient perfusion cell cultures were initiated by seeding polycarbonate shake flasks (Corning, Product Numbers 431,143, 431,144, 431,145, and 431,147, USA) ranging from 125 to 1000‐mL at 0.2–0.4 × 10^6^ cells/mL. All used shake flasks are unbaffled unless otherwise specified. Flasks were agitated in a 5% CO_2_ incubator at 37 °C, 75% relative humidity, 120–300 RPM agitation with a 25‐mm orbit, with a working volume of 20% (v/v) of the flask maximum total volume. Once cell densities reached 1.5–2.5 × 10^6^ cells/mL, full media exchange was done by centrifugation in conical tubes at 300×g for 5 min. Samples of cell suspension taken before centrifugation were used for cell counts and key metabolites (glucose, lactate, and ammonia) measurements. The cell pellets were disrupted by gentle tapping of the tube and resuspended in BalanCD CHO Growth A media supplemented with a pre‐defined % (v/v) of 0.8X BalanCD CHO Feed4 (Fujifilm Irvine Scientific, USA). Medium is supplemented with 0.8X BalanCD CHO Feed4 to avoid nutrient depletion given the high cell densities achieved. Cell cultures were then placed back in incubator conditions until the next sampling and media exchange. Cell cultures were ended when important drops in cell viability were observed to avoid seeding production bioreactors with cells of declining viability below 90%.

### 2.3 Bioreactor cell culture

Both cell culture modes, standard seeding density (SSD) and ultra‐high seeding density (UHSD), were performed in 1‐L BioFlo120 (Eppendorf, Germany) under fed‐batch mode with an initial volume of 650 mL. Cells were cultivated in BalanCD CHO Growth A (Fujifilm Irvine Scientific, USA) supplemented with 50 μM MSX, 0.1% (w/v) Kolliphor P188 (BASF, Germany) and fed with 0.8X BalanCD CHO Feed4 (Fujifilm Irvine Scientific, USA) according to an in‐house developed bolus feed schedule every 2–3 days based on platform work to optimize for cell growth and productivity. The culture pH was controlled at 7.0 ± 0.2 through carbon dioxide (CO_2_) sparging and the addition of an in‐house 4% (w/v) sodium hydroxide and 9% (w/v) bicarbonate base solution. Cultures were agitated using a 3‐blade 45° pitched‐blade impeller with a 5.8‐cm diameter at an estimated volumetric power input (P/V) of 35 W/m^3^. The latter relates to volume, mixing speed, impeller diameter, and impeller number.^1^ Dissolved oxygen levels (DO) were controlled through 25 mL/min surface air gassing and a cascade of air and oxygen sparging. Cascade aeration first used air on demand to control DO at the desired setpoint until reaching a maximum air flowrate of 2 mL/min, after which the air flowrate was supplemented with pure oxygen as needed. At 0 days post induction (DPI), cell cultures were induced with 2 μg/mL of cumate (4‐Isopropylbenzoic acid, Ark Pharm Inc., USA). Cell cultures were terminated when measured cell viability reached below 70%.

The SSD cell cultures were seeded at 0.4 × 10^6^ cells/mL at −3 DPI (three days prior to induction day). Temperature was maintained at 37 °C until 2 DPI after which temperature was down‐shifted to 32 °C until the end of the culture. Temperature shift was done because of its positive impact on protein production and culture longevity.^30^ The DO was maintained at 60% of air saturation. The UHSD cell cultures were seeded at 15 × 10^6^ cells/mL at 0 DPI. The induction was performed at the time of cell seeding, thus eliminating the growth phase in the bioreactors. The temperature was maintained at 32 °C throughout the culture. The DO was maintained at 40% of air saturation.

### 2.4 Gas transfer rates and shear stress estimations in shake flasks

The OTR of cell cultures in shake flasks was measured with the Transfer‐rate Online Monitoring (TOM) system (Kuhner Inc., Switzerland). The cell culture conditions during OTR measurement are those described in semi‐continuous transient perfusion methods. The flask headspace aeration flowrate was set to 16 mL/min, and OTR measurements were performed over 105 min, with 35 min of measuring time and 6 min of high flow flushing out time.^31^ During the remaining 64 min of the cycle, the system operates under surface aeration mode, which aims to mimic aeration conditions of shake flasks in an incubator with 5% CO_2_ and 75% relative humidity at 37 °C. The maximum OTR of the shake flask was measured using the sulfite system.^32^ A 0.35 M sodium sulfite solution (≥99%; Sigma, USA) in 0.012 M phosphate buffer prepared with deionized water adjusted to pH 8 was used (Na_2_HPO_4_ ≥99% purity; NaH_2_PO_4_ ≥99% purity; Sigma, USA). The reaction was catalyzed by 10^−7^ M cobalt sulfate (≥ 99% purity; Sigma, USA). The maximum OTR measurements with the sulfite system (cell‐free system) were conducted in the same flask sizes, fill volumes, and incubating conditions as the cell cultures they replicate.

The average energy dissipation rate (Ɛ_∅_) can be calculated (Equation 2) from agitation rate (n), flask diameter (d), liquid volume (V_L_), and the modified Newton number (Ne′) as seen in Equation 3.^33^ The modified Newton number is estimated in unbaffled shake flasks using Reynold's number (Equation 3).^34^ The Reynold's number can be calculated from Equation 4 including the liquid density (ρ), the agitation rate (n), the flask diameter (d), and dynamic fluid viscosity (η).^33^ In unbaffled shake flasks, the maximum energy dissipation rate (Ɛ_max_) is equal to the average energy dissipation rate for Reynolds numbers below 60,000 and can be calculated from Equation 5 for Reynolds numbers above 60,000 (d_o_ is orbital shaking diameter).^35^ From Ɛ_max_ and the kinematic viscosity (ν), the Kolmogorov length (λ_K_) can be calculated (Equation 6).

(2) $$\varepsilon_{\emptyset }=Ne^{\prime }\times \frac{n^{3}\times d^{4}}{V_{L}^{2/3}}$$

(3) Ne′=70Re−1+25Re−0.6+1.5Re−0.2

(4) Re=ρ×n×d2η

(5) $$\varepsilon_{\max}=\frac{0.1{\left(\pi \times n\times d\right)}^{3}}{1.11\times d_{0}^{0.18}\times d^{-0.11}\times n^{0.44}\times V_{L}^{0.34}}$$

(6) $$\lambda_{K}=\sqrt[{4}]{\frac{\nu^{3}}{\varepsilon_{\max}}}$$

### 2.5 Sample handling

Cell counts and viability measurements were done by means of trypan blue exclusion staining using a Cedex MS20C Automated Cell Counter (Innovatis, Germany). Sample supernatants obtained by centrifugation at 5000×g for 5 min were used for glucose, lactate, ammonia, monoclonal antibody (mAb), and amino acid concentration measurements. Glucose, lactate, and ammonia concentrations were measured by colorimetric assays using Vitros350 (Ortho‐Clinical Diagnostics, USA). Amino acid measurements were done following the AccQ‐Tag Ultra Derivatization Kit (Waters Corporation, USA) protocol using the Acquity H‐Class UPLC system (Waters Corporation, USA). The mAb titer determination was done by Protein A HPLC. The sample supernatants were filtered in MultiScreen HV 96‐well filtration plates at 1500×g for 2 min (Durapore®, 0.45 μm, Millipore, USA) to remove cellular debris. The HPLC was performed using a 2695/2996 HPLC system (WATERS Corporation, USA) with a Protein A cartridge (POROS® A20 column, 2.1 mm D × 30 mm H, Thermo Fisher Scientific, Part# 2‐1001‐00). A phosphate buffer saline solution without calcium and magnesium (Cat. No. SH30028.03, Cytiva, USA) is injected into the column before loading the samples at 2 mL/min. The column was washed with 1 mL, 10 column volumes, to remove unbound species and cell culture media components. A 0.15 M NaCl elution buffer at pH 2.0 was used for 1 min to detach the antibodies from the column. The mAb product was detected by UV at 280 nm with a typical error below 10%.

## 3 RESULTS AND DISCUSSION

### 3.1 Time‐of‐action study

**FIGURE 1:**
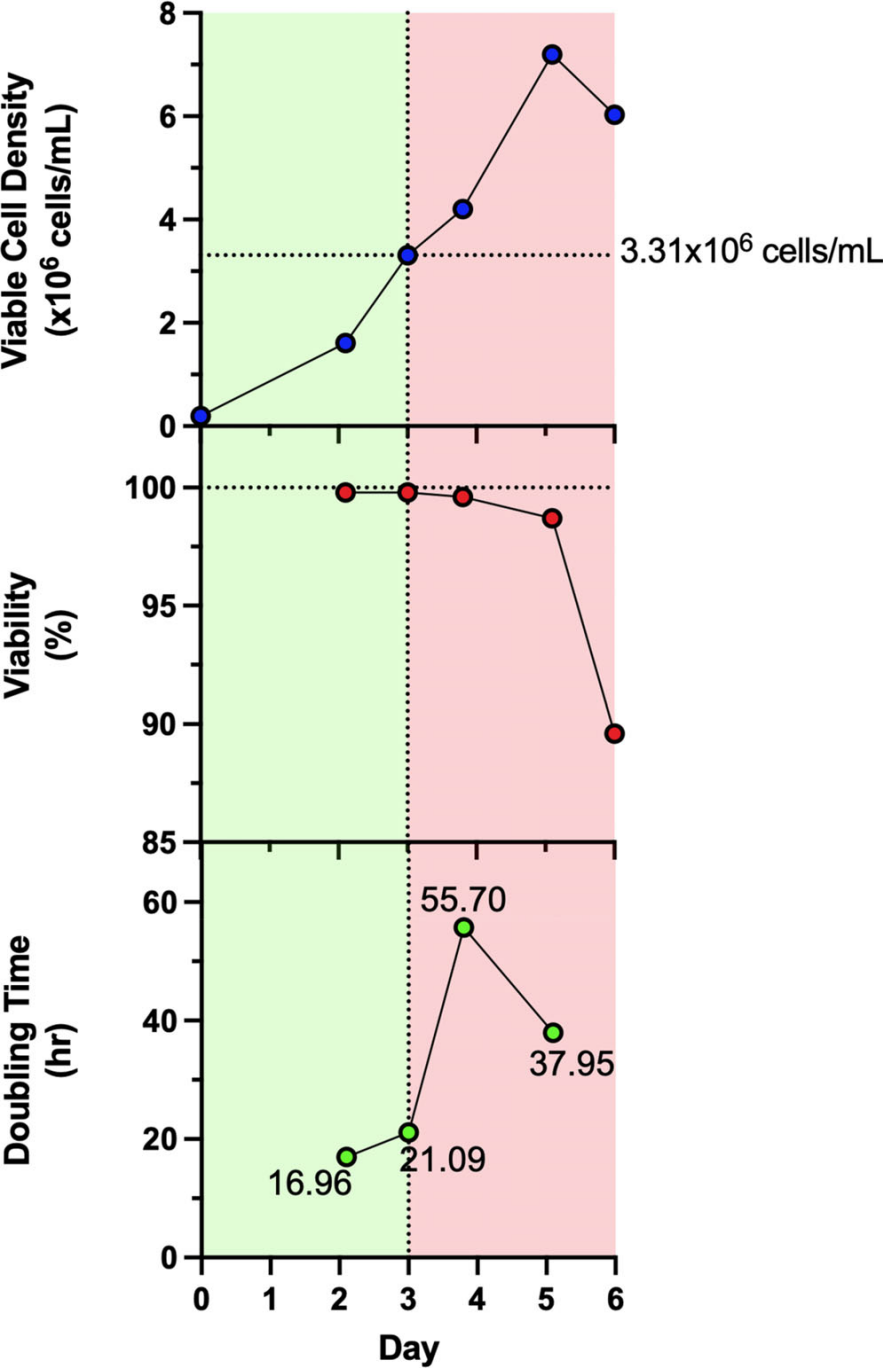
Time‐of‐action study for determination of beginning of media replacement for semi‐continuous transient perfusion in shake flasks. Viable cell density (blue), cell viability (red), and cell doubling time (green) of a 50 mL batch cell culture in 250‐mL shake flasks. Green area represents cell densities at which viability is maintained at >98% and when cells are under exponential growth as shown by the corresponding doubling times.

A time‐of‐action study was performed to determine a suitable timing for the first media exchange of semi‐continuous transient perfusion in shake flasks. A batch cell culture was performed in a 250‐mL baffled shake flask (Corning, USA) at a working volume of 50 mL and an initial viable cell density of 0.2 × 10^6^ cells/mL. The viable cell density (VCD), cell viability, and cell doubling time were monitored daily to identify at which cell density either the cell viability begins to drop or doubling times increase indicating the culture is no longer in exponential growth (Figure 1). Historical cell doubling times during exponential growth for this cell line were on average 18.6 ± 2.0 h. (*n* = 46). A VCD of 3.31 × 10^6^ cells/mL was selected as the maximum density at which media exchange will be initiated. A doubling time of 21.09 h was observed which falls within 2 standard deviations of the historical mean. As such, going forward, media exchange was started at VCD of 1.5–2.5 × 10^6^ cells/mL to ensure media exchange is begun before cells enter late exponential and plateau phase of cell growth. The cell density of 3.31 × 10^6^ cells/mL being at the end of the exponential growth phase is in line with observations from literature where batch CHO cells cultures in shake flasks typically reach the late exponential phase by 4–6 × 10^6^ cells/mL.^36^, ^37^, ^38^, ^39^, ^40^

### 3.2 Effect of media exchange frequency

**FIGURE 2:**
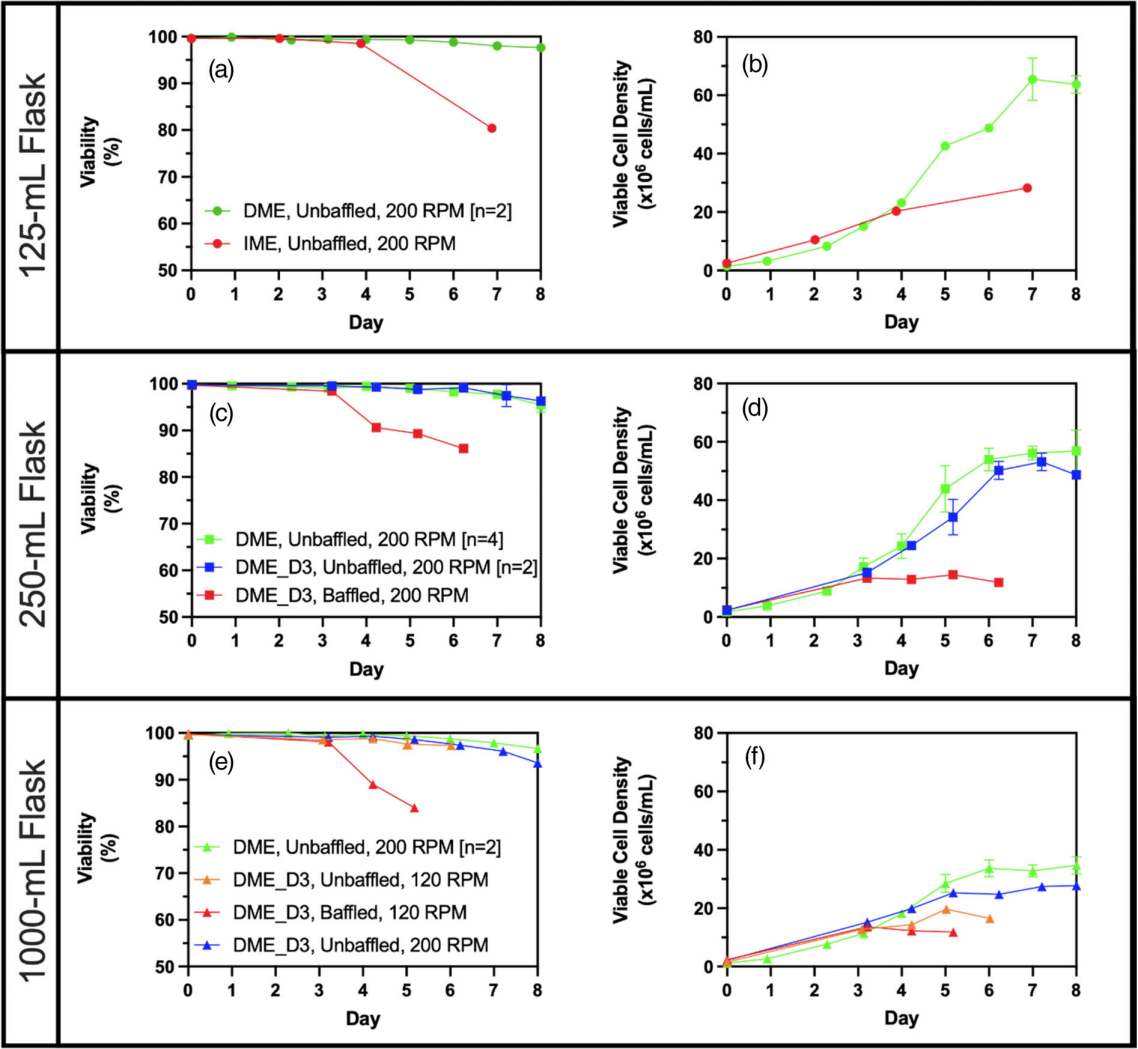
Effect of Media Exchange Frequency performed in shake flasks. (a) Viable cell density and (b) cell viability of daily media exchange (DME, green −●‐) and cell passages replaced by media exchange (IME, red −●‐) in unbaffled 125‐mL shake flasks; (c) Viable cell density and (d) cell viability of daily media exchange in unbaffled flasks (DME, green −■‐), daily media exchange beginning at day 3 in unbaffled flasks (DME_D3, blue −■‐), and daily media exchange beginning at day 3 in baffled flasks (DME_D3, red −■‐) for 250‐mL shake flasks; (e) Viable cell density and (f) cell viability of daily media exchange in unbaffled flasks agitated at 200 RPM (DME, green −▲‐), daily media exchange beginning at day 3 in unbaffled flasks agitated at 200 RPM (DME_D3, blue −▲‐), daily media exchange beginning at day 3 in unbaffled flasks agitated at 120 RPM (DME_D3, orange −▲‐), and daily media exchange beginning at day 3 in baffled flasks agitated at 120 RPM (DME_D3, red −▲‐) for 1000‐mL shake flasks. DME refers to daily media exchange; IME refers to the intermittent media exchange of cell passages replaced by media exchange; DME_D3 refers to media exchange at day 0 and daily media exchange beginning on day 3. Error bars of duplicates represent minimum and maximum values while error bars of quadruplicates represent standard deviation of replicates.

During cell expansion, cell culture passages were performed every 2–3 days to ensure cells are kept in the exponential growth phase. The first media exchange experiment in shake flasks simply replaced cell dilution with media exchange. In this case, cell dilution and media exchange serve the same purpose: removal of metabolic waste such as lactate and ammonia and replenishment of nutrients such as glucose, key amino acids, and other substrates. The 125‐mL unbaffled shake flask was first used to test the effect of intermittent media exchanges (IME) (performed on days 0, 2, 4, and 7) starting at a VCD of 1.5–2.5 × 10^6^ cells/mL (Figure 2a and b). From day 0 to 4, cell viability is maintained at a >98% value and a VCD of 20.3 × 10^6^ cells/mL is achieved, which represents a doubling time of 30.66 h. This prolongation in doubling time due to cell biomass accumulation is expected and has previously been observed and modeled for perfusion cultures.^5^, ^41^ On day 7, the cell growth rate had strongly decreased, with a doubling time of 47.88 h, and cell viability had decreased to 80.4%. This drop in cell viability can be explained by glucose depletion at day 7. The specific glucose consumption rate of this cell line during the cell growth phase is ~1 pmol/cell/day. With this consumption rate and an estimated VCD at day 7, a glucose concentration of ~80 mM is required. However, high glucose concentrations (>55 mM) have been reported to decrease the cell growth rate.^42^ To solve this issue and maintain glucose concentrations low without depletion, media exchange was done daily (DME) in a 125‐mL unbaffled shake flask, beginning media exchange on day 0 at a cell concentration of 1.5–2.5 × 10^6^ cells/mL (Figure 2a,b). With daily media exchange (DME), glucose concentrations were successfully maintained between 6 and 45 mM, cell viability was shown to be high at >98%, and a peak VCD of 65.5 ± 7.2 × 10^6^ cells/mL (*n* = 2) was achieved on day 7 at a doubling time of 30.1 ± 1.2 h (*n* = 2). Although no cell growth is observed between day 7 and 8, no significant loss of viability was detected.

The semi‐continuous transient perfusion cell expansion process with daily media exchange (DME) was performed in 250‐mL shake flasks (Figure 2 c,d) and in 1000‐mL shake flasks (Figure 2e,f). Maximum cell concentrations of 56.1 ± 7.1 × 10^6^ cells/mL (*n* = 4) at day 7 and 33.7 ± 2.9 × 10^6^ cells/mL (*n* = 2) at day 6, respectively, were achieved with >98% cell viability. As OTR in shake flasks is a function of flask size,^19^ the reduced maximum VCD in larger flasks was suspected to be due to oxygen transfer limitations (as confirmed further in this study). Unbaffled shake flasks were thus switched for baffled flasks, which are known to increase OTR.^43^ Initially, to reduce media consumption of the process, media exchange was not performed on day 1 and 2 when VCD is low enough such that glucose would not be depleted in this time frame, as seen in the 125‐mL shake flask cultures. However, these conditions resulted in early cell death after day 3 in both 250 and 1000‐mL baffled flasks (Figure 2c–f). As a result of early cell death in baffled flasks, maximum VCD was significantly lower than in daily media exchanged unbaffled flasks. Premature cell decline observed in baffled shake flask cultures without media exchange on day 1 and 2 was attributed to shear stress rather than nutrient limitation. This conclusion was supported by experiments using unbaffled flasks without media exchange on days 1 and 2, conducted in 250‐mL shake flasks (Figure 2c,d) and 1000‐mL shake flasks (Figure 2e,f). This is consistent with literature where cell death was observed in baffled shake flasks after prolonged exposure to a higher shear stress environment.^44^ Maximum cell concentrations of 53.2 ± 3.0 × 10^6^ cells/mL (*n* = 2) and 27.4 × 10^6^ cells/mL, respectively, at day 7 were achieved with >93% cell viability. To further demonstrate the impact of oxygen limitations, media exchange cell culture was performed in 1000‐mL flasks agitated at a lower orbital agitation rate of 120 RPM (Figure 2e,f). Although cell viability was maintained at >98%, a maximum VCD of only 19.7 × 10^6^ cells/mL was achieved on day 5. This observation is in line with expected oxygen transfer limitations.

**FIGURE 3:**
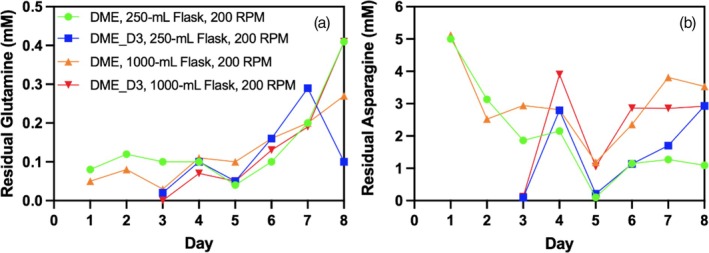
L‐Glutamine and L‐Asparagine kinetic profile in 250‐ and 1000‐mL shake flask. (a) Residual glutamine and (b) residual asparagine of semi‐continuous transient perfusion cell cultures with daily media exchange (DME) in 250‐mL flasks (green −●‐) and 1000‐mL flasks (orange −▲‐) and daily media exchange with day 1 and 2 omitted (DME_D3) in 250‐mL flasks (blue −■‐) and 1000‐mL flasks (red −▼‐).

From the 20 protein‐forming amino acids measured, only asparagine (Asn) and glutamine (Gln) were observed to be depleted during semi‐continuous transient perfusion in shake flasks with media exchange (Supplemental Figure 1). In both daily media exchange (DME) and daily media exchange with days 1 and 2 omitted (DME_D3) performed in 250 and 1000‐mL shake flasks, glutamine levels remain low throughout cell culture, showing slightly higher levels toward the end of the culture (Figure 3a). Low glutamine levels are typical for CHO‐GS cells, which can synthesize glutamine from glutamate and ammonia due to the expression of the glutamate synthetase enzyme. It is worth mentioning the low endogenous GS expression in CHO cells in general. Consequently, no additional glutamine supplementation was performed in the media and feed formulated for CHO‐GS cells, as previously described.^27^ This small quantity of glutamine produced is rapidly consumed by the cells, leading to a low residual level detected transiently in the cell culture. Moreover, the frequent media exchange also prevents its accumulation. Asparagine has been observed to be depleted on day 3 in both 250 and 1000‐mL shake flasks when media exchange is omitted on days 1 and 2 (Figure 3b). Both glutamine and asparagine, which can be used interchangeably by cells, are particularly important during the early exponential cell growth phase for their contribution of carbon and nitrogen sources into the TCA cycle.^45^ Asparagine, which enters the TCA cycle through its conversion to aspartate by asparaginase, is then converted to oxaloacetate catalyzed by glutamic‐oxaloacetic transaminase 1,^46^ contributing to the total carbon flux to the TCA cycle.^47^ Although glutamine and asparagine can be used interchangeably as nutrient sources for the TCA cycle, in the DME_D3 semi‐continuous transient perfusion cell culture, both of these amino acids are rapidly depleted on day 3 as no nutrients are supplied on days 1 and 2. Furthermore, asparagine was shown to be crucial in CHO‐GS cell growth and its lack could result in cell growth arrest.^48^ Growth arrest of asparagine‐depleted DME_D3 cell cultures can also be confirmed by the slower rate of increase in OTR, which is expected to be proportional to VCD, before day 3 and then restored after media exchange on day 3 (Supplemental Figure 2).^31^ Asparagine depletion is also observed in 250‐mL flasks for both DME and DME_D3 media exchange schedules due to elevated cell concentrations. However, glutamine, which can be used concomitantly with asparagine, is not completely depleted on day 5.

### 3.3 Effect of flask size on semi‐continuous transient perfusion

**FIGURE 4:**
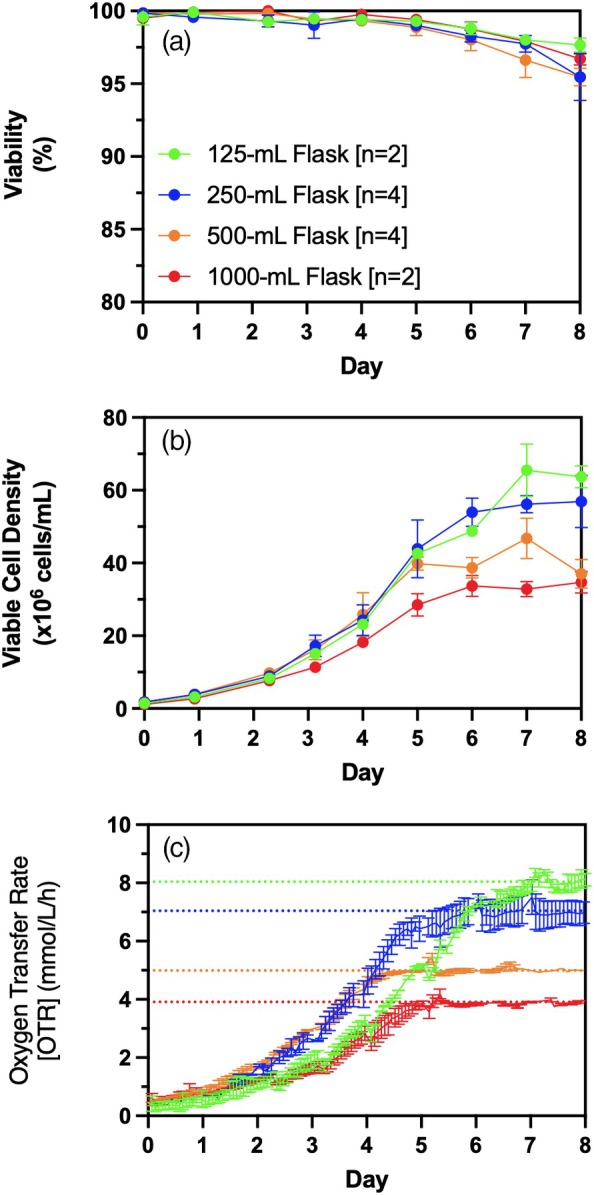
Effect of flask size on cell growth and OTR profile. (a) Cell viability, (b) viable cell density, and (c) oxygen transfer rate (OTR) of cell cultures undergoing complete daily media exchange. Horizontal bars indicate the maximum OTR observed for each flask size (c). Cell cultures were performed in unbaffled flask 125‐mL (green −●‐), 250‐mL (blue −●‐), 500‐mL (orange −●‐), and 1000‐mL (red −●‐) at fill volumes of 20% and agitated at 200 RPM. Error bars of duplicates represent minimum and maximum values while error bars of quadruplicates represent standard deviation of replicates. OTR measurements for 250 and 500‐mL flasks were done in duplicates [*n* = 2].

The impact of flask size on maximum VCD and oxygen transfer limitations was evaluated using 125, 250, 500, and 1000‐mL shake flasks, each at 20% of maximum nominal volume (Figure 4). The OTR was monitored throughout the semi‐continuous transient perfusion cultures using the TOM system. To maximize VCD, cultures were agitated at 200 RPM in unbaffled flasks with daily media exchange starting from day 0, based on previous frequency of media exchange optimization studies. Cell viability is maintained at >98% for all flask sizes while in exponential cell growth (Figure 4). The observed maximum VCD and OTR are inversely proportional to the flask size (Table 1). The maximum VCD is proportional to the maximum OTR, indicating that the specific respiration rate of the cells at the maximum cell density achieved in each flask is the same at 2.8 ± 0.2 pmol/cell/day. This specific respiration rate value falls within the expected range of 1–10 pmol/cell/day for CHO cells.^25^, ^49^ The observed OTR curve throughout cell culture follows the expected tendency for oxygen‐limiting cell cultures, where OTR increases during cell growth until reaching a plateau and maintaining this plateau for some time.^50^

**TABLE 1:** Maximum observed viable cell densities and oxygen transfer rates of semi‐continuous transient perfusion cell cultures with daily media exchange in 125‐, 250‐, 500‐, and 1000‐mL shake flasks at a fill volume of 20% and an agitation rate of 200 RPM.

Flask size	Maximum observed viable cell Density (×10^6^ cells/mL)	Maximum observed oxygen transfer rate (mmol/L/h)
125‐mL	65.5 ± 7.2 (*n* = 2)	8.04
250‐mL	56.1 ± 7.1 (n = 4)	7.04
500‐mL	46.7 ± 3.9 (n = 4)	4.99
1000‐mL	33.7 ± 2.9 (n = 2)	3.91

Equation 7 allows estimation of maximum OTR in shake flasks as a function of osmolarity (Osmol), agitation rate (n), operating volume (V_L_), flask diameter (d), orbital shaking diameter (d_0_), operating pressure (p_R_), and oxygen molar fraction (y_O2_).^19^ Although this equation has an outstandingly small error of ±5 mmol/L/h for bacterial cell cultures, it does not allow determining if the observed maximum OTR is due to oxygen transfer limitations in the present mammalian cell cultures. This correlation was created for use with bacteria, which have a much higher oxygen consumption rate than mammalian cells. Since the measured maximum OTRs of this study fall in the lower portion (4–8 mmol/L/h) of the range of applicability of this correlation (0–80 mmol/L/h), the error makes it impossible to determine if different flask sizes theoretically result in significantly different maximum OTRs.

(7) $${\mathrm{OTR}}_{\max}=\begin{array}{l} 3.72\times 10^{-7}\times {\text{Osmol}}^{0.05}\times n^{\left(1.18-\frac{\text{Osmol}}{10.1}\right)}\times V_{L}^{-0.74}\times d_{0}^{0.33}\\ \times d^{1.88}\times p_{R}\times y_{O2} \end{array}$$

A sulfite system was used to measure maximum OTR to confirm that oxygen transfer limitations, rather than other factors such as nutrient depletion or metabolic waste accumulation, caused the observed maximum OTR in media exchanged cell cultures (Figure 4c). To do this, sodium sulfite is used to simulate rapid oxygen consumption as it is converted to sodium sulfate by catalysis of cobalt sulfate (II) at sufficiently high rates such that the OTR is considered the rate limiting step.^51^, ^52^ The maximum OTR measured with the sulfite system for 125‐, 250‐, 500‐, and 1000‐mL flasks at a 20% fill volume was inversely proportional to flask size (Table 2 with raw data presented in Supplemental Figure 3). The maximum OTR measurements obtained with the sulfite system (cell‐free) were compared to the maximum OTR observed in the biological system, which are the maximum OTRs achieved during daily media exchange in shake flasks agitated at 200 RPM (Figure 4c). These maximum OTRs (cell‐free sulfite system) represent a 2%–26% difference compared to the maximum OTRs observed in the biological system when doing the daily media exchange (Table 2).

**TABLE 2:** Maximum oxygen transfer rates observed in biological systems and measured in sulfite systems (cell‐free) for 125‐, 250‐, 500‐, and 1000‐mL unbaffled shake flasks at 20% fill volume agitated at 200 RPM. Maximum OTR measurements in the sulfite system were done in triplicates (OTRmax, sulfite, *n* = 3). Maximum OTR measurements in the biological system were done in duplicates (OTRmax, (bio, *n* = 2).

	Flask size			
	125‐mL	250‐mL	500‐mL	1000‐mL
OTR_max,sulfite_ (mmol/L/h)	9.6 ± 0.1	6.9 ± 0.3	6.1 ± 0.5	4.9 ± 0.1
OTR_max,bio_ (mmol/L/h)	8.04	7.04	4.99	3.91
Difference (%)	18.7	1.8	22.2	25.8
OTR_max,bio_/OTR_max,sulfite_	0.84	1.02	0.82	0.80

There exists a difference between the measured maximum OTR of the sulfite system and the biological system. This difference can be in part attributed to the differences in osmolarity and diffusion coefficients of the solutions.^19^ Although differences between culture media and the sulfite system at different concentrations of sodium sulfite exist, a concentration of sodium sulfite of 0.35 M was shown to have comparable gas transfer kinetics to culture media.^32^ As such, a 0.35 M concentration of sodium sulfite was used to reduce the difference between the biological maximum OTR and the sulfite system maximum OTR. Furthermore, to measure the maximum OTR with the sulfite system, the reaction kinetics must be adjusted to a “non‐accelerated” reaction regime, which allows only negligible reaction of oxygen and sulfite in the liquid side boundary layer. This allows for most of the oxygen to be consumed in the bulk liquid, similarly to biological systems where oxygen transfer occurs at the liquid film formed on the surface of the flask and oxygen consumption occurs in the bulk liquid. Using a 10^−7^ M concentration of the cobalt sulfate (II) catalyst allows for a “non‐accelerated” reaction regime with the sulfite system.^32^ Thus, through the general trend of the OTR curve and the sulfite system measurements of maximum shake flask OTR, it was shown that the maximum cell density of this CHO cell line was attained in shake flasks due to oxygen transfer limitations.

### 3.4 Effect of orbital agitation speed on cell growth and OTR

**FIGURE 5:**
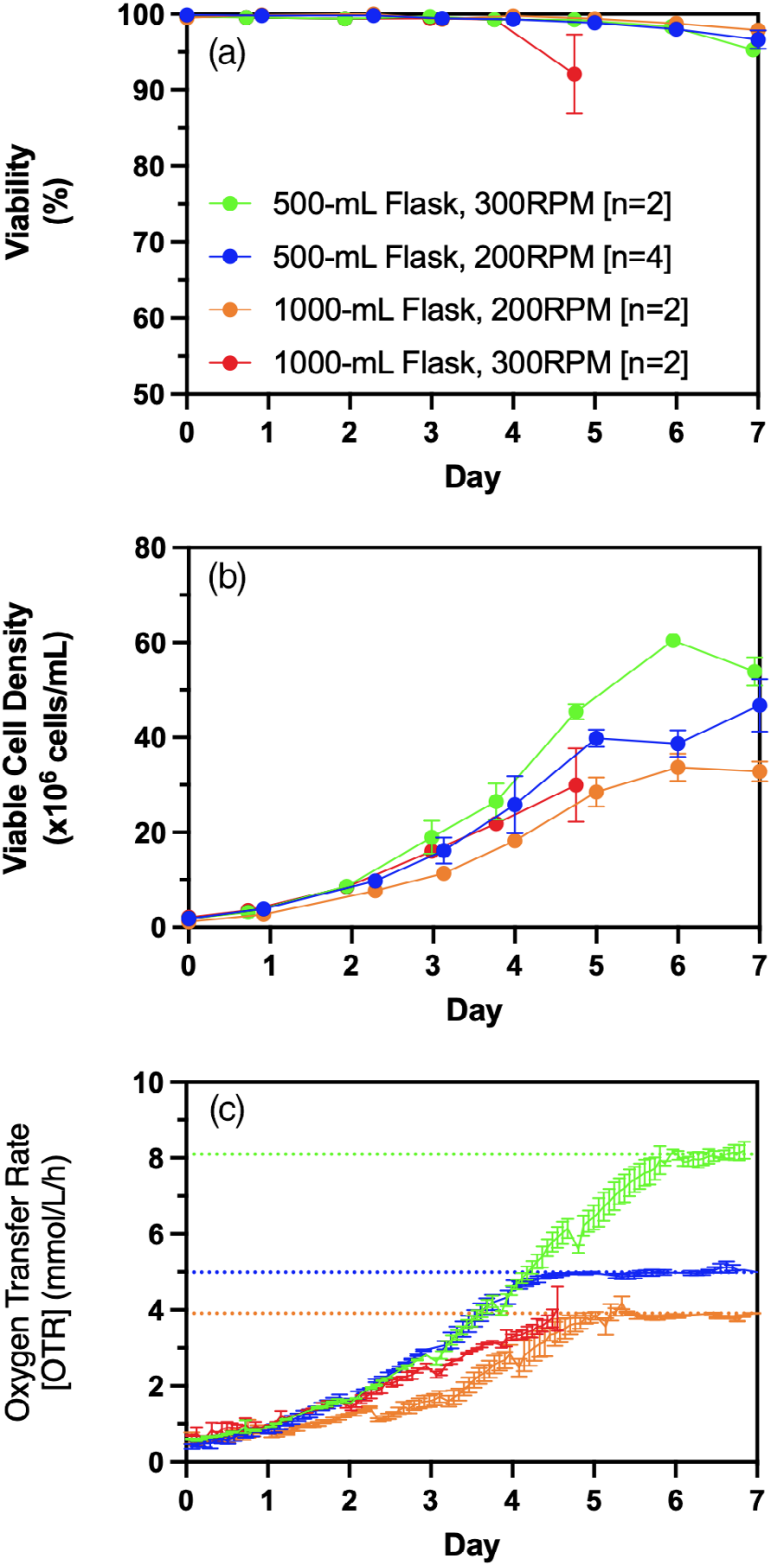
Effect of orbital shaking speed on cell growth and OTR. (a) Cell viability, (b) viable cell density, and (c) oxygen transfer rate (OTR) of cell cultures undergoing complete daily media exchange. Cell cultures were performed in unbaffled flasks at shaking frequencies of 200 and 300 RPM: 500‐mL at 300 RPM (green −●‐), 500‐mL at 200 RPM (blue −●‐), 1000‐mL at 200 RPM (orange −●‐), and 1000‐mL at 300 RPM (red −●‐). Error bars of duplicates represent minimum and maximum values and error bars of quadruplicates represent standard deviation of replicates. OTR measurements for 500‐mL flasks at 200 RPM (blue curve, c) were done in duplicate [*n* = 2] due to technical issues.

As it was shown that the maximum sustainable VCD by oxygen transfer was achieved, reaching greater VCD in shake flasks by semi‐continuous transient perfusion requires increasing the maximum OTR of the system. The OTR of shake flasks can be increased by the addition of baffles, which induces greater turbulence.^52^ However, the addition of baffles was shown to lead to an early decrease in cell viability (Figure 2). The maximum OTR of a shake flask can also be increased by decreasing operating volume, increasing shaking diameter, or increasing shaking frequency (Equation 7). Shaking frequency was selected to increase maximum OTR, as shaking diameter has a lesser effect on maximum OTR.^19^ On the other hand, reducing operating volume would reduce the total quantity of cells produced by semi‐continuous transient perfusion, which goes against the purpose of cell expansion for seeding of a benchtop bioreactor. Shaking frequency was increased from 200 RPM to 300 RPM for 500‐ and 1000‐mL flasks for semi‐continuous transient perfusion with daily media exchange (Figure 5). In 500‐mL flasks, the increase in shaking rate resulted in an observed maximum OTR of 4.99 to 8.10 mmol/L/h. This increase in maximum OTR allowed for an improvement of maximum VCD from 46.7 ± 3.9 × 10^6^ cells/mL (*n* = 4) at 200 RPM to 60.5 ± 0.2 × 10^6^ cells/mL (*n* = 2) at 300 RPM. However, in the case of the 1000‐mL flask, the increase in agitation rate did not result in a rise in maximum VCD and maximum OTR, as cell viability began to decrease after day 4. The cell culture in the 1000‐mL flask at 300 RPM was terminated early due to an observed early decline in cell viability.

An increase in shaking speed results in increased shear stress in shake flasks.^43^ Furthermore, shake flasks with larger volume, and thus larger flask diameter, are subjected to higher levels of shear stress at the same shaking frequency when compared to smaller flasks.^33^, ^53^ The average energy dissipation rates increase by 1.6‐fold when shake flask sizes are varied from 500‐ to 1000‐mL and by 3.2‐fold when agitation rates are increased from 200 to 300 RPM (Table 3). The Kolmogorov length represents the size of the smaller eddies formed in mixing. When the size of these eddies is smaller than cell size, cell damage may occur.^54^ In 1000‐mL shake flasks, a Kolmogorov length of 13.6 μm is estimated, which is below that of the average CHO cell diameter of 15 μm. Thus, cell death in the 1000‐mL flask at 300 RPM is most likely due to prolonged exposure to elevated shear stress conditions.

**TABLE 3:** Reynolds numbers (Re), average (Ɛ_∅_) and maximum (Ɛ_max_) energy dissipation rates, and Kolmogorov lengths $\left(\lambda_{K}\right)$ in 500 and 1000‐mL shake flasks at 20% fill volume and different agitation rates.

Flask size	500‐mL	500‐mL	1000‐mL	1000‐mL
Agitation rate (RPM)	200	300	200	300
Re	49,138	73,707	78,921	118,382
$\varepsilon_{\emptyset }$ (W/kg)	0.38	1.2	0.54	1.7
$\varepsilon_{\max}$ (W/kg)	0.38	6.1	3.6	10
$\lambda_{K}$ (μm)	30.8	15.4	17.6	13.6

### 3.5 Effect of semi‐continuous transient perfusion on cell doubling time and productivity

**FIGURE 6:**
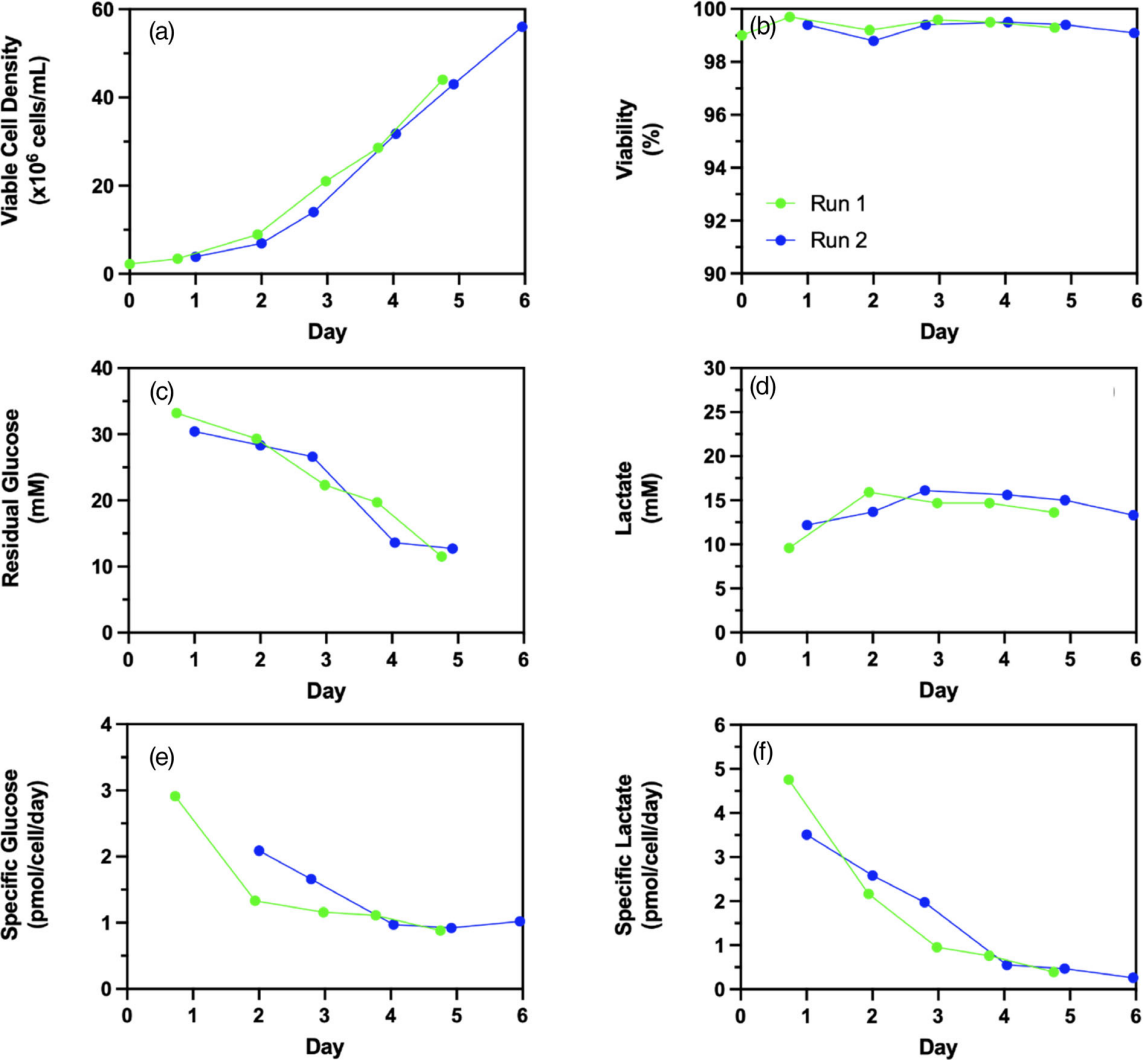
Effect of daily medium replacement on shake flask cell growth performance. (a) Viable cell density, (b) cell viability, (c) residual glucose, (d) lactate, (e) cell specific glucose consumption, and (f) cell specific lactate production for Run 1 (green −●‐) and Run 2 (blue −●‐) of daily media exchange in 125‐mL unbaffled shake flasks at 20% fill volume agitated at 300 RPM. Run 1 refers to the semi‐continuous transient perfusion cell expansion from thawed cells. Run 2 refers to semi‐continuous transient perfusion cell expansion from Run 1 cells at day 5 after reaching >40 × 10^6^ cells/mL.

To determine the effect of medium replacement on cell growth rate, daily media exchange was performed on cells grown in a 125‐mL shake flask at 20% fill volume (25 mL) agitated at 300 RPM from 2 × 10^6^ cells/mL (labeled “Run 1”). When reaching 40 × 10^6^ cells/mL, cells were diluted back to 2 × 10^6^ cells/mL and daily media exchange was performed once again until cells had reached over 40 × 10^6^ cells/mL (labeled “Run 2”). The cell viability was maintained at >98% throughout both Run 1 and Run 2 cell expansions (Figure 6b). The growth curves of Run 1 and Run 2 overlap (Figure 6a) indicating that semi‐continuous transient perfusion in shake flasks through complete daily media exchange has no short‐term impact on cell growth rate. Furthermore, residual glucose and lactate levels as well as specific glucose consumption and lactate production were comparable in Run 1 and Run 2 of semi‐continuous transient perfusion cell expansion (Figure 6c–f).

**FIGURE 7:**
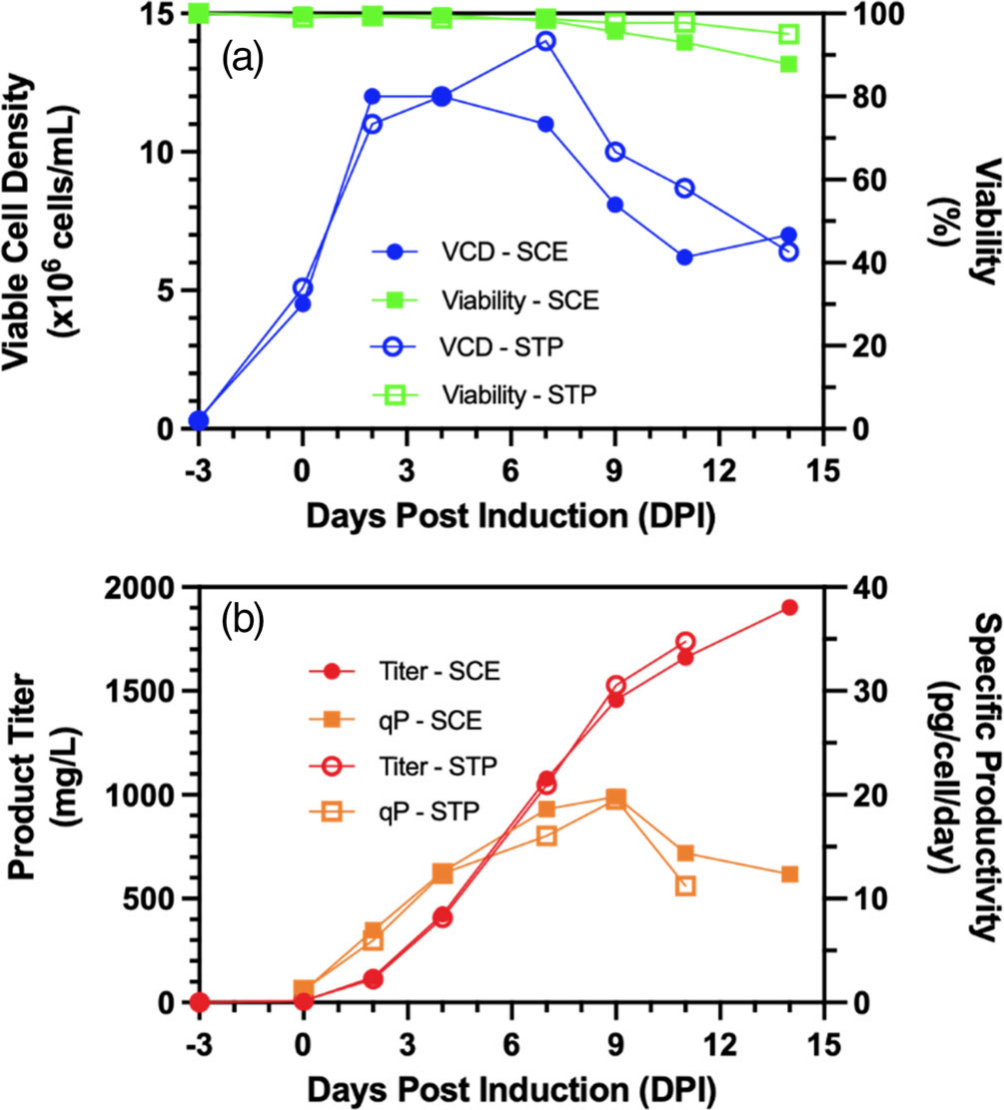
Effect of daily medium replacement cell expansion on shake flask fed‐batch production. (a) Viable cell density & cell viability and (b) volumetric product titer & cell specific productivity of cell cultures in 125‐mL baffled shake flasks seeded from standard cell expansion (SCE) and semi‐continuous transient perfusion (STP).

To determine the effect of daily media exchange on culture productivity, two cell cultures were performed in shake flasks under identical conditions but with different seed trains. One flask was seeded using standard cell expansion (SCE) where cells were maintained in exponential growth by diluting the cells to maintain viable cell density between 0.2 to 3 × 10^6^ cells/mL. A second flask was seeded with cells coming from semi‐continuous transient perfusion (STP) where cells were subjected to daily media exchange from 2.2 × 10^6^ cells/mL on day 0 until day 5 when the cells reached >40 × 10^6^ cells/mL. Cells were diluted to 2 × 10^6^ cells/mL and daily media exchange was performed until day 11 when the cells reached 56 × 10^6^ cells/mL (Figure 6a). Shake flask cell cultures seeded from SCE and STP both had comparable growth rates during the growth phase until 2 DPI. The decrease in VCD throughout the production phase (2–14 DPI) is comparable for both cultures (Figure 7a). Cell specific productivities were similar throughout cell cultures seeded with both SCE and STP (Figure 7b). As a result of comparable specific productivity and VCD, volumetric product titers were similar for cultures seeded from both cell expansion methods. These results show that the STP has no observable impact on cell growth and glycolytic metabolism (Figure 6) and it does not impact cell growth and viability as well as cell specific and volumetric mAb productivity (Figure 7).

### 3.6 Improvement of increased seeding density

**FIGURE 8:**
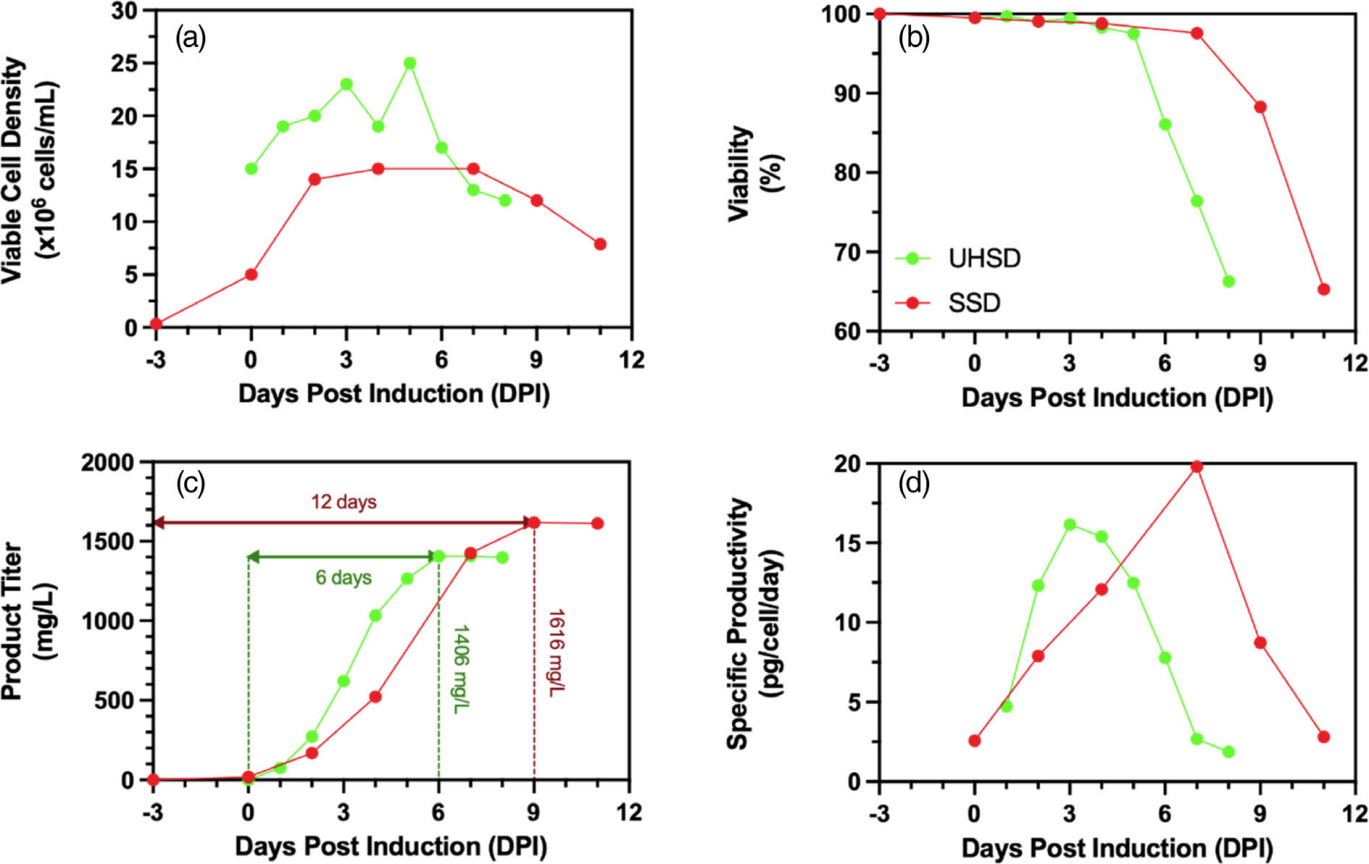
Effect of ultra‐high seeding density (UHSD) on fed‐batch bioreactor production performance. (a) Viable cell density, (b) cell viability, (c) volumetric product titer, and (d) cell specific titer of ultra‐high seeding density (UHSD) (green −●‐) and standard seeding density (SSD) (red −●‐) cell cultures in 1‐L benchtop bioreactors.

To demonstrate that STP in shake flasks through daily medium exchange can be used to seed benchtop bioreactors at high cell density, a 1‐L vessel was inoculated at ultra‐high seeding density (UHSD) (15 × 10^6^ cells/mL) from a STP N‐1 seed train. A second 1‐L benchtop bioreactor was seeded at standard seeding density (SSD) (0.3 × 10^6^ cells/mL) from a SCE N‐1 seed train. The UHSD process began at 15 × 10^6^ cells/mL at induction while the SSD process was still only at ~5 × 10^6^ cells/mL at induction. The SSD reached ~15 × 10^6^ cells/mL 2 days later than the UHSD process (Figure 8a). Both processes were maintained at >95% viability for ~5 days (Figure 8b). The UHSD process achieved earlier peak cell viability but had an earlier decline in cell viability, which is typical of high‐density seeding processes as previously observed in high seeding density studies.^6^, ^55^ Attaining high VCD earlier in the cell culture allowed for a shorter cell culture in the UHSD process compared to the SSD process. The high‐density seeding process produced 1206 mg/L of mAb in 6 days while the standard process produced 1616 mg/L in 12 days. This represents only a 25.4% loss of product concentration for a process that is 50% shorter in timeline. As such, the space–time yield (STY) of the UHSD process was 201 mg/L/day while the STY of the SSD process was only 135 mg/L/day, representing a 49.3% increase in STY. The shorter culture time in the UHSD process is achieved by bypassing the pre‐induction growth phase (−3 to 0 DPI) and starting the production phase (0 to 2 DPI) at a higher cell density, leading to an earlier productivity peak. Further optimization of UHSD cultures can be done, which is outside the scope of this study. These future optimizations can include enhanced feeding regimens, supplement additions such as short chain fatty acids like sodium butyrate, valeric acid, or valproic acid, which have been shown to arrest cell growth and promote specific cell productivity,^6^ real‐time glucose measurements to enable a low glucose level control strategy, and key amino acid supplementation.^8^

## 4 CONCLUSION

The prime objective of this study to seed a benchtop bioreactor at ultra‐high cell densities using shake flasks in N‐1 precultures to reduce process costs and enable process simplicity was achieved. Additionally, the improvements of the UHSD process were demonstrated by comparing to a SSD process, where the UHSD process had a 49.3% reduction in space–time yield before process optimization.

The development of the N‐1 seed train aims to mimic transient perfusion, such as is done commonly with ATF/TFF perfusion systems, to reach viable cell densities capable of seeding a benchtop bioreactor at 15 × 10^6^ cells/mL. Semi‐continuous transient perfusion done through media exchange in shake flasks was used to approximate transient perfusion in a bioreactor. The first step in this development was to determine the necessary frequency of media exchange. The results demonstrated that at lower frequencies (every 2–3 days), total glucose consumptions would become high enough that glucose would be depleted, or high enough glucose concentrations would be necessary that would become inhibitory to cell growth. Daily media exchange from day 0, beginning at 1.5–2.5 × 10^6^ cells/mL, was necessary to achieve the highest viable cell density. In fact, when media exchange was omitted on days 1 and 2 to reduce media consumption, lower maximum viable cell densities were achieved due to nutrient limitations.

It was then shown, through OTR measurements of cell cultures using the TOM instrument (Kuhner Shaker Inc.), that maximum cell densities are achieved when oxygen becomes a limiting nutrient. Measured maximum OTR using the sulfite system confirms that cell cultures reached viable cell densities that are oxygen‐limiting in 125‐, 250‐, 500, and 1000‐mL shake flasks at 20% fill volume agitated at 200 RPM. An increase in agitation rate from 200 to 300 RPM for the 500‐mL flasks resulted in an increase of maximum OTR from 4.99 to 8.10, allowing an improvement in maximum VCD of 29.6%. However, for the 1000‐mL flask, raising the agitation rate from 200 to 300 RPM resulted in early cell death and thus a lower maximum VCD. The early cell death is attributed to the higher shear stress present in larger shake flasks with higher agitation rates. Since cell viability is not negatively impacted at 200 RPM in 1000‐mL shake flasks but is impacted at 300 RPM, the agitation rate of this flask could be further optimized (between 200 and 300 RPM) to find a maximum OTR that does not impact cell viability.

Semi‐continuous transient perfusion in shake flasks was successfully used as a N‐1 preculture for the ultra‐high density seeding of 1‐L benchtop bioreactors, thus circumventing the need for expensive and specialized perfusion equipment. The use of ultra‐high seeding density (15 × 10^6^ cells/mL) in benchtop bioreactor cell cultures resulted in 87% of the volumetric product titer obtained with standard seeding densities (0.3 × 10^6^ cells/mL). However, the UHSD cell culture had a 50% shorter culture time when compared to the SSD cell culture. The much shorter culture time paired with comparable volumetric product titers allowed for a 49.3% increase in space–time yield. This increased STY is mainly due to the elimination of the growth phase performed prior to the N production. When this growth phase time is considered, the difference in space–time yield is much smaller due to the achieved final titers being comparable. Nonetheless, with further optimization, literature shows that higher titers are achievable in high density seeding cultures.^3^ Finally, using N‐1 perfusion seed train allows for shorter usage of the N production bioreactor, enabling a better equipment flexibility and a high asset turnover ratio.

## AUTHOR CONTRIBUTIONS

**Lucas Lemire:** Conceptualization; methodology; data curation; investigation; writing – original draft. **Sebastian‐Juan Reyes:** Formal analysis; writing – review and editing. **Yves Durocher:** Supervision; resources; formal analysis. **Robert Voyer:** Formal analysis; supervision; resources. **Olivier Henry:** Supervision; resources; project administration; validation; writing – review and editing; funding acquisition; formal analysis. **Phuong Lan Pham:** Supervision; resources; project administration; validation; writing – review and editing; funding acquisition; formal analysis.

## CONFLICT OF INTEREST STATEMENT

The authors declare no conflicts of interest.

## Supporting information


**Data S1:** Supporting Information.

## Data Availability

The data that support the findings of this study are available from the corresponding author upon reasonable request.
