# Supplementary material for: N‐1 semi‐continuous transient perfusion in shake flask for ultra‐high density seeding of CHO cell cultures in benchtop bioreactors
Source: Biotechnol Prog. 2025 Apr 3;41(5):e70029. doi: 10.1002/btpr.70029 (PMC12531934; doi:10.1002/btpr.70029)
Supplement: Supplementary file 1 — Data S1: Supporting Information. [file BTPR-41-e70029-s001.docx]

# SUPPLEMENTARY DATA


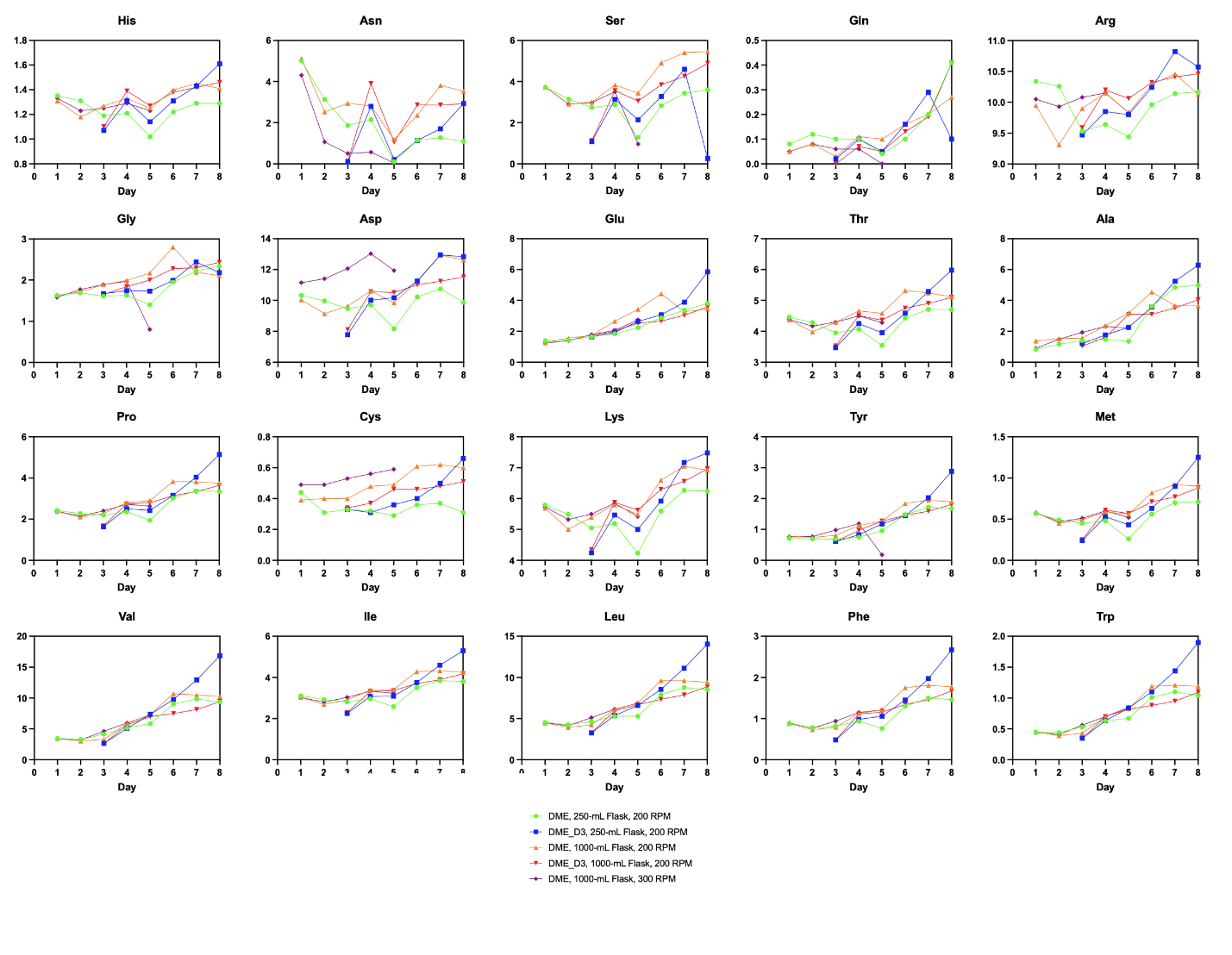


Supplemental Figure 1: Amino acids measured throughout semi-continuous transient perfusion in shake flasks for daily media exchange (DME) and daily media exchange daily except for days 1 and 2 (DME_D3) media exchange frequency.


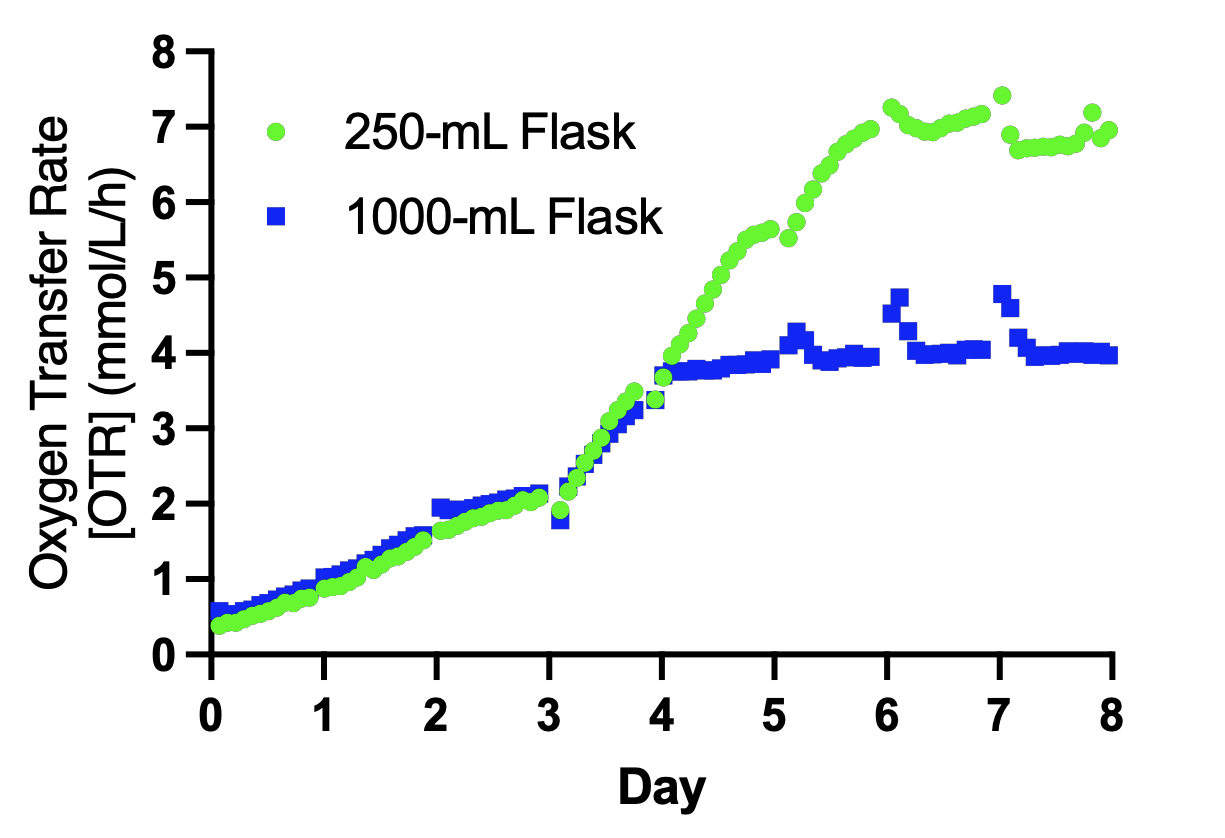


Supplemental Figure 2: Oxygen transfer rate of semi-continuous transient perfusion cultures in 250-mL and 1000-mL shake flasks with daily media exchange except day 1 and 2 (DME_D3) agitated at 200 RPM.


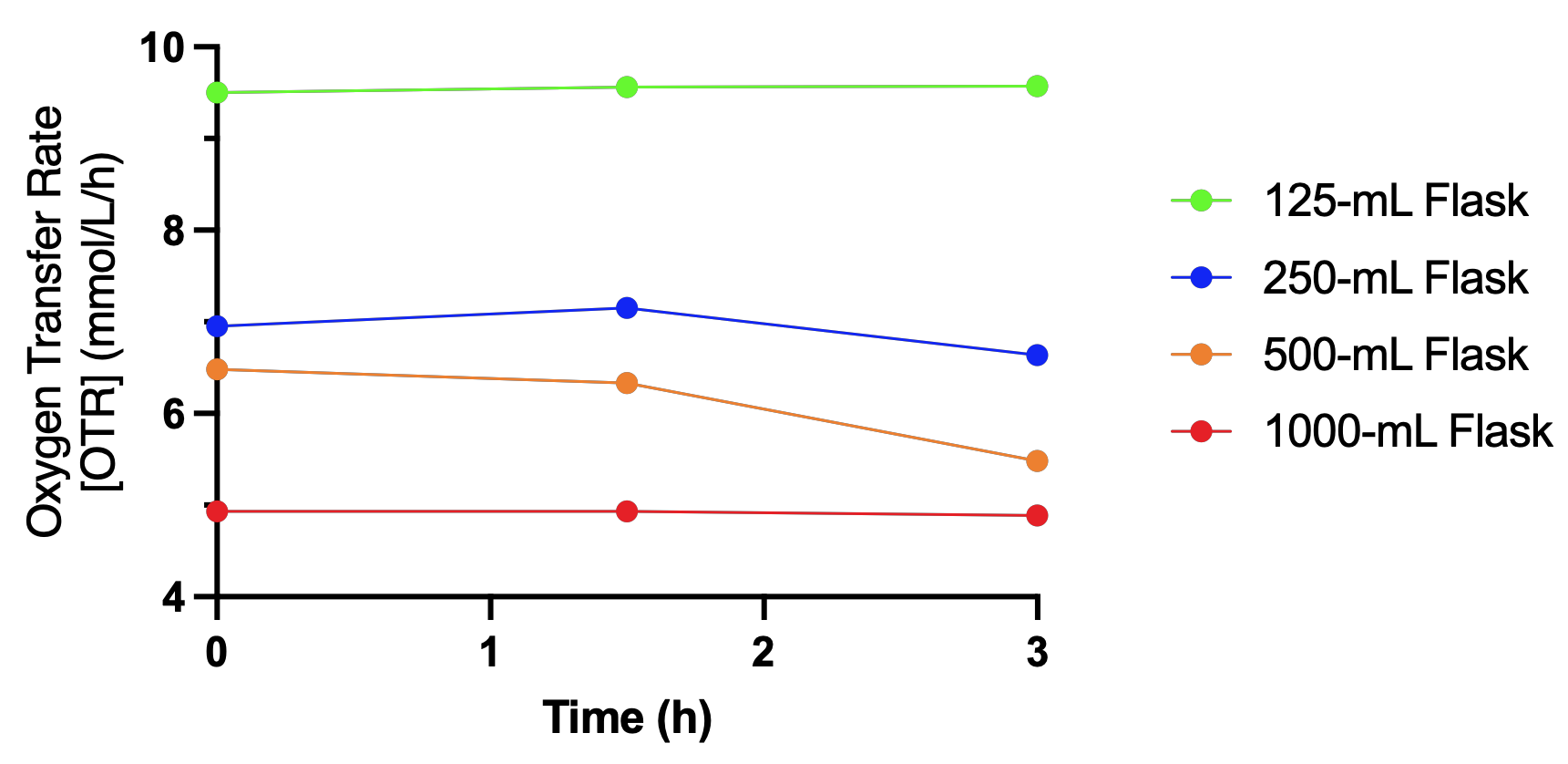


Supplemental Figure 3: Maximum oxygen transfer rate measured using the sulfite system in 125-mL, 250-mL, 500-mL, and 1000-mL flasks at 20% fill volume agitated at 200 RPM.
